# Successful Operation for Aneurism of the Posterior Aural Artery
*Mr. Fletcher's Med. and Chirurg. Illustrations.


**Published:** 1831

**Authors:** 


XIII.

### SUCCESSFUL OPERATION FOR ANEURISM OF THE POSTERIOR AURAL ARTERY.*

"HESTER CHAMBERS, aged thirty-four years, was admitted into the General

* Mr. Fletcher's Med. and Chirurg. Illustrations.

Hospital here, on account of an ulceration behind the left ear, attended with a most excruciating pain of the head, and the left side of the throat and neck; likewise with very great pain in swallowing. These pains had much of the neuralgic character, followed the course of the nerves, were periodical in violence, though seldom entirely absent.

There was a small tumour, apparently of the skin, about the size of half a very large nutmeg, seated an inch and a half obliquely above the mastoid process on the affected side, the projecting surface of which was ulcerated, and the ulceration extended a little farther than its basis: it had been of near seven years standing, and was accustomed now and then to bleed freely from its surface, and once it discharged, she said, nearly half-a-pint of blood. For three months before her admission to the Hospital it had not bled; and since the bleeding had ceased, the pains of the head and neck had come on, which at first were confined to one side of the head only, but afterwards were extended very generally over its surface.

Medicines, bleeding, both topical and general, blisters, &c., had been tried for her relief, with little or no benefit. The ulceration of the tumour and integument were healed, but the pain nevertheless continued, or even increased in violence. She had applied to several practitioners, and had been told her complaint was a cancer.

Upon examining the neighbourhood of the little tumour behind the ear very minutely, a small oblong and soft swelling was observed, which was continued from the base of the tumour, and which distinctly pulsated. After repeated examinations it was clear that there was an aneurism, or dilatation of a small branch of the occipital artery which runs up behind the ear; and though it was not so clear how this should occasion the woman's sufferings, yet it was obvious that the tumour and the pains were connected, and it was, therefore, resolved to tie up the dilated artery. By making one pressure at the lower edge of the mastoid process, and another about an inch and a half higher up on the scalp, the woman's sufferings were instantly suspended. The artery was laid bare between these two points, and at each of them a ligature was passed underneath it,—the needle carried close to the bone, and a strong ligature made. The artery cut into was found to be dilated to the size of a goose-quill, as it quitted the tumour. The woman instantly lost all her pain, and could immediately swallow with ease.

About a month after the operation she was discharged, perfectly well."

Thirteen months afterwards this patient returned, having suffered for the last ten weeks from a violent pain in the same side of the head, extending from the site of the former incision upwards towards the vertex. No enlargement of any vessel could be discovered, but pressure applied a little above the seat of the pain immediately suspended it. Across this part there was made an incision two or three inches in length, continued through part of the fascia of the temporal muscle. The pain instantaneously left her, but in the evening she became affected with severe head-ache, and was extremely giddy and thirsty. Deafness, in some degree, succeeded, with various nervous symptoms, and, though the pain in the site of the incision was lost, it returned in the spot where the first operation had been performed. The pulse was always frequent, sometimes small, no treatment was of service, and the patient was recommended to leave the house. At length she begged that some operation might be performed, and one of a rather extraordinary character was adopted. An incision was made, a portion of the scalp and pericranium half an inch or an inch in breadth *removed* from the mastoid process upwards, for a space of three inches, and the cranium *bored* with a terebra as far as the diploë. The former pain left her immediately, but in the evening noises in the head, giddiness, &c. occurred, and before the wound was healed, violent pain was complained of in a line close to the posterior and inferior boundary of the mastoid process. Here a deep incision was made, and some of the fibres of the trapezius cut. The pain instantly ceased and in less than a month the patient was discharged well, and she still remains so, although there is no sensibility in the greater part of the scalp on the affected side of the head.

This is one of those extraordinary cases to which one scarcely knows what to say. The end may be considered as consecrating the means, success as stamping the character of the treatment. Suppose, however, that from such an operation as was here attempted, matter were to form upon the dura mater and occasion the patient's death, what would the opinion of the world be then? And suppose that this operation, severe as it was, and fraught with danger as it might have been, had proved unsuccessful and inoperative;—what might be the criticisms of the harsh and the illiberal? Mr. Brodie mentions a case in which a surgeon performed some such an operation on the scalp for a similar disease; the patient died in consequence of the formation of matter on the dura mater and secondary deposites. Another consideration is this, that nervous pains do occasionally subside without the assistance of art, certainly without that of surgery, and not infrequently resist the most scientific as well as the most severe operations.

Looking at the present case from every point of view, and impartially considering the practice, we cannot avow that we think it safe, or that we should feel inclined to adopt it. Still this must be looked on as merely an opinion, and Mr. Fletcher has on his side of the question, an argument of no mean value, the fortunate issue of the experiment.

